# Supplementary material for: Live-imaging rate-of-kill compound profiling for Chagas disease drug discovery with a new automated high-content assay
Source: PLoS Negl Trop Dis. 2021 Oct 11;15(10):e0009870. doi: 10.1371/journal.pntd.0009870 (PMC8530327; doi:10.1371/journal.pntd.0009870)
Supplement: S1 Text — (DOCX) [file pntd.0009870.s001.docx]

ATGGACTCGACTGAAAATGTGATAAAGCCATTTATGCGTTTTAAAGTCCACATGGAGGGATCGGTTAATGGGCACGAATTTGAGATCGAGGGCGTTGGGGAGGGTAAGCCATACGAAGGTACTCAAACGGCCAAATTGCAAGTGACTAAAGGAGGACCACTTCCGTTTGCTTGGGACATCCTTTCACCGCAATTTTTCTACGGAAGCAAAGCATACATCAAACACCCTGCGGACATACCCGACTACCTGAAACAATCCTTTCCAGAGGGATTTAAATGGGAGAGGGTCATGAACTTTGAAGACGGCGGTGTGGTGACTGTTACCCAGGACAGCTCGCTCCAAGATGGCACTTTGATCTACCATGTCAAATTTATTGGCGTGAACTTCCCGTCGGATGGCCCTGTGATGCAAAAAAAGACCCTCGGGTGGGAACCTTCAACCGAACGGAATTACCCTAGGGATGGTGTTCTTAAGGGCGAGAATCACATGGCGCTCAAGCTGAAGGGGGGTGGTCATTACCTCTGCGAGTTCAAATCCATTTACATGGCTAAGAAACCCGTGAAGTTGCCTGGATATCATTACGTGGATTATAAACTCGATATAACCTCCCACAATGAGGATTACACCGTGGTTGAGCAATATGAACGCGCGGAAGCGCGGCACCATTTGTTCCAGTAG
