# Supplementary figures and images for: Live-imaging rate-of-kill compound profiling for Chagas disease drug discovery with a new automated high-content assay
Source: PLoS Negl Trop Dis. 2021 Oct 11;15(10):e0009870. doi: 10.1371/journal.pntd.0009870 (PMC8530327; doi:10.1371/journal.pntd.0009870)

**
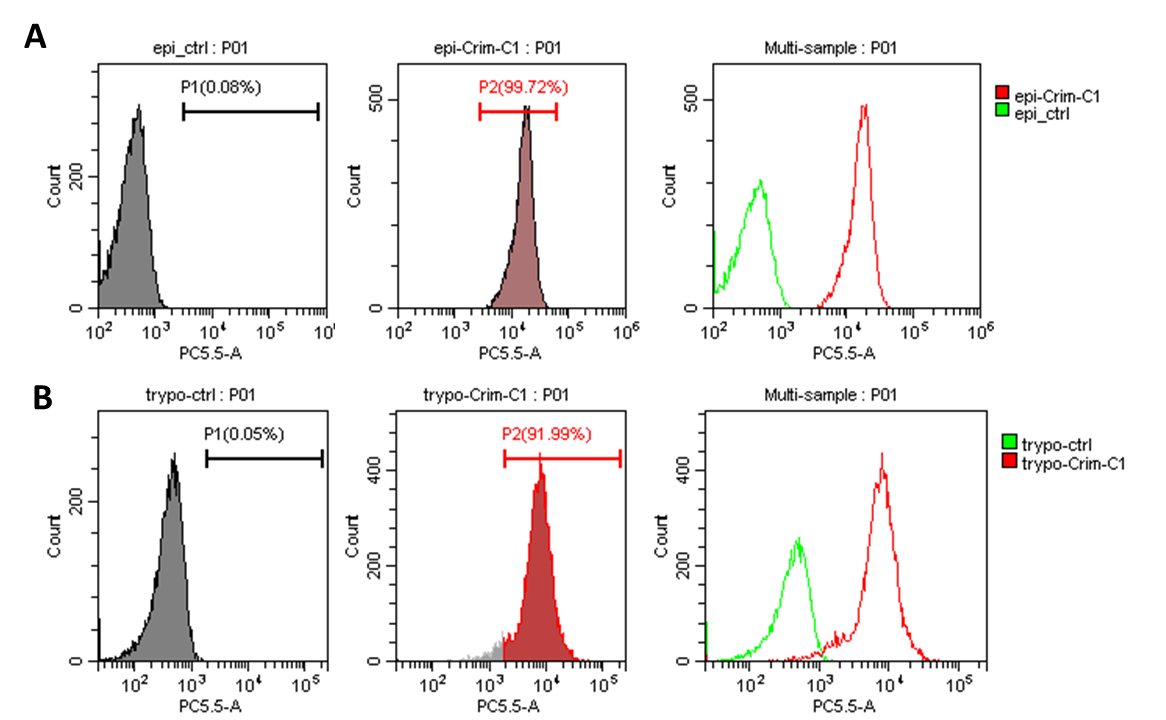
**

Supplement: S1 Fig — A: comparison of control wild type epimastigotes and Tc-X10/7-E2Crimson clone C1 epimastigotes. Over 99% of the Tc-X10/7-E2Crimson-expressing epimastigote population is positive for E2Crimson. B: comparison of control wild type trypomastigotes and Tc-X10/7-E2Crimson clone C1 trypomastigotes. 92% of the Tc-X10/7-E2Crimson-expressing trypomastigotes population is positive for E2Crimson. (DOCX) [file pntd.0009870.s002.docx]

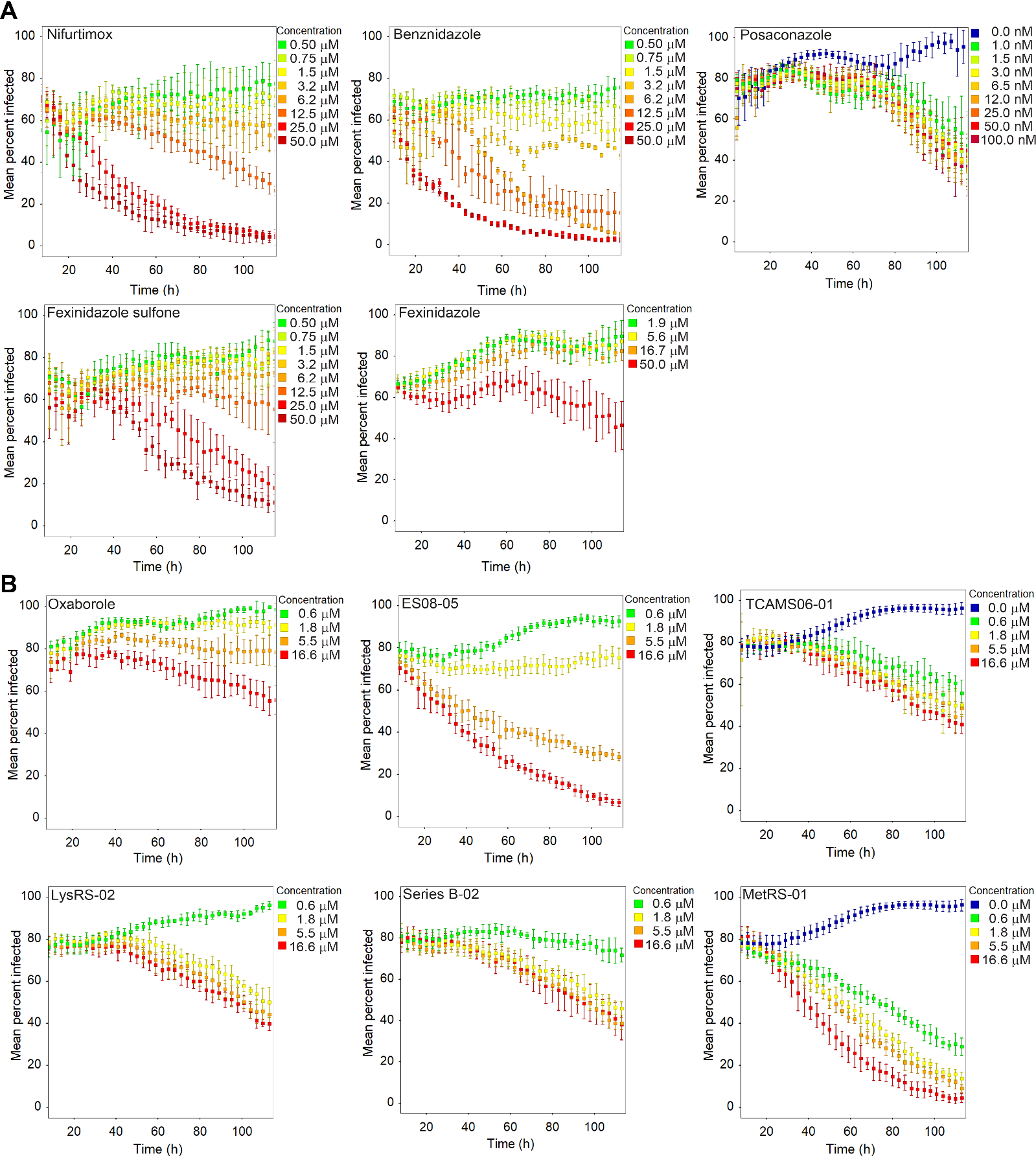

Supplement: S2 Fig — Live rate-of-kill curves for A) reference compounds and B) representative compounds from each hit series plotted as percent infected host cells versus time. Error bars in all panels represent standard deviations for 6 technical replicates. For compounds were all concentrations showed parasite reduction the DMSO vehicle control plot is also included (blue). (DOCX) [file pntd.0009870.s003.docx]

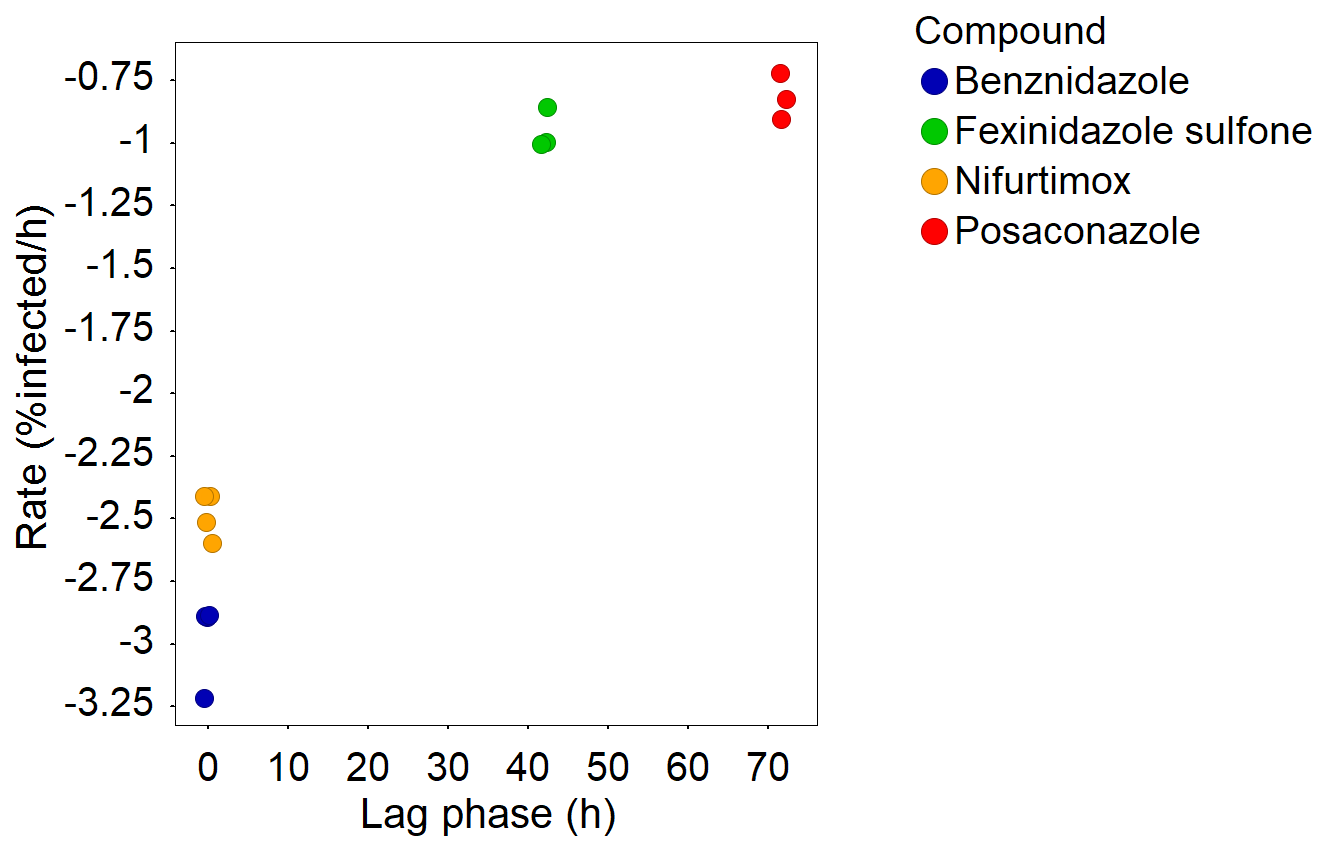

Supplement: S3 Fig — (DOCX) [file pntd.0009870.s004.docx]

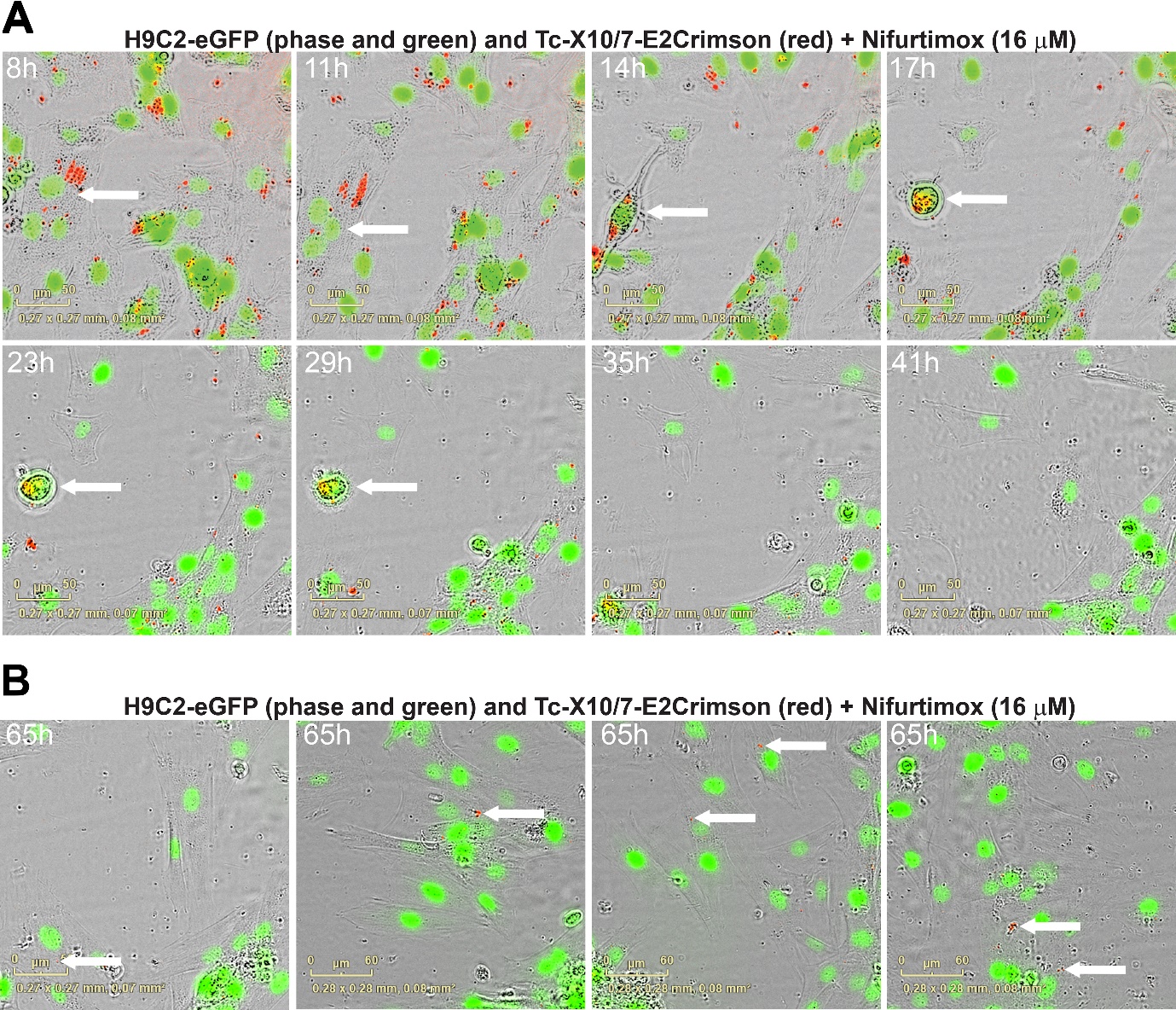

Supplement: S4 Fig — Images from rate-of-kill experiments illustrating cell death of infected host cells (A) and small number of residual parasites after 65 hours of treatment with 16μM Nifurtimox (B). Green fluorescence: H9C2 host cell nuclei, red fluorescence: T. cruzi parasites. All images in panel A are for the same field of view. Arrows in panel A point to the same dying cell in each image. Arrows in panel B point to residual parasites. (DOCX) [file pntd.0009870.s005.docx]
