# Supplementary material for: Live-imaging rate-of-kill compound profiling for Chagas disease drug discovery with a new automated high-content assay
Source: PLoS Negl Trop Dis. 2021 Oct 11;15(10):e0009870. doi: 10.1371/journal.pntd.0009870 (PMC8530327; doi:10.1371/journal.pntd.0009870)
Supplement: S1 Table — (DOCX) [file pntd.0009870.s006.docx]

| **Compound** | **Replicate** | **Slope (%infected/h)** | **SD** | **R2** | **Lag phase (h)** |
| --- | --- | --- | --- | --- | --- |
| **Posaconazole** | **1** | **-0.81** | **0.010** | **0.99** | **72** |
|  | **2** | **-0.92** | **0.014** | **0.98** | **72** |
|  | **3** | **-0.72** | **0.021** | **0.93** | **72** |
| **Fexinidazole sulfone** | **1** | **-0.98** | **0.015** | **0.99** | **42** |
|  | **2** | **-1.00** | **0.006** | **1.00** | **42** |
|  | **3** | **-0.86** | **0.040** | **0.91** | **42** |
| **Benznidazole** | **1** | **-2.90** | **0.190** | **0.89** | **0** |
|  | **2** | **-3.20** | **0.089** | **0.98** | **0** |
|  | **3** | **-2.90** | **0.051** | **0.99** | **0** |
|  | **4** | **-2.90** | **0.090** | **0.98** | **0** |
| **Nifurtimox** | **1** | **-2.60** | **0.046** | **0.97** | **0** |
|  | **2** | **-2.50** | **0.051** | **0.95** | **0** |
|  | **3** | **-2.40** | **0.062** | **0.94** | **0** |
|  | **4** | **-2.40** | **0.041** | **0.97** | **0** |
| **ES08** | **1** | **-0.61** | **0.006** | **0.99** | **0** |
|  | **2** | **-0.60** | **0.011** | **0.96** | **0** |
|  | **3** | **-0.49** | **0.007** | **0.98** | **0** |
|  | **4** | **-0.44** | **0.007** | **0.98** | **0** |
|  | **5** | **-0.71** | **0.013** | **0.97** | **0** |
| **TCAMS06** | **1** | **-0.44** | **0.009** | **0.97** | **23** |
|  | **2** | **-0.42** | **0.012** | **0.95** | **23** |
|  | **3** | **-0.41** | **0.008** | **0.97** | **23** |
| **B series** | **1** | **-0.51** | **0.007** | **0.98** | **32** |
|  | **2** | **-0.56** | **0.005** | **0.99** | **32** |
| **LysRS** | **1** | **-0.42** | **0.008** | **0.97** | **44** |
|  | **2** | **-0.46** | **0.007** | **0.98** | **44** |
| **MetRS** | **1** | **-0.77** | **0.015** | **0.96** | **0** |
|  | **2** | **-0.58** | **0.008** | **0.98** | **0** |
|  | **3** | **-0.55** | **0.010** | **0.97** | **0** |
|  | **4** | **-0.57** | **0.005** | **0.99** | **0** |
| **Oxaborole SCYX-6759** | **1** | **-0.30** | **0.005** | **0.97** | **28** |
|  | **2** | **-0.36** | **0.010** | **0.92** | **27** |
|  | **3** | **-0.35** | **0.008** | **0.95** | **26** |
