# Supplementary material for: Live-imaging rate-of-kill compound profiling for Chagas disease drug discovery with a new automated high-content assay
Source: PLoS Negl Trop Dis. 2021 Oct 11;15(10):e0009870. doi: 10.1371/journal.pntd.0009870 (PMC8530327; doi:10.1371/journal.pntd.0009870)
Supplement: Attachment — Submitted filename: PNTD-D-21-00718 Reviewers comments.docx [file pntd.0009870.s011.docx]

Reviewer's Responses to Questions

<b>Key Review Criteria Required for Acceptance?</b></br></br> As you describe the new analyses required for acceptance, please consider the following:</br></br>

<b>Methods</b></br></br>

-Are the objectives of the study clearly articulated with a clear testable hypothesis stated?</br> -Is the study design appropriate to address the stated objectives?</br> -Is the population clearly described and appropriate for the hypothesis being tested?</br> -Is the sample size sufficient to ensure adequate power to address the hypothesis being tested?</br> -Were correct statistical analysis used to support conclusions?</br> -Are there concerns about ethical or regulatory requirements being met?</br></br>

Reviewer #1: General comments:

- Objectives were clearly stated

- Study design addresses partially the rate of kill data to identify fast killing compounds. It is challenging to compare across compounds given single concentration used in the study. A complete dose response would have given better clarity for comparison across the compounds.

We thank the reviewer for raising this important point and have now included an additional figure (Figure S2) with dose response data. This includes 8 concentrations for the clinical compounds and 4 concentrations for the NCEs.

- Study fails to justify why only fast acting cpds need to be prioritized.

We do not argue that only fast acting compounds should be progressed, just that in a triaging process rate-of-kill should be considered, with preference for fast acting compounds. The clarify our thinking we have added to the discussion: “Rate-of kill data can be used to prioritise faster acting compounds that may offer a higher chance of delivering a short treatment regimen in the clinic. Clearly, prioritisation is multi-factorial and other factors need to be considered as well, including toxicity liabilities and ability to clear persister parasites, which may be independent of the rate-of-kill.”

- claims of rate of kill data provided being helpful for PKPD modeling and combination strategy lacks justification and data to support these claims.

It is beyond the scope of our work to include data with regard to PKPD modelling, but we agree with the reviewer that we do not explain the use of rate-of-kill data for PKPD modelling sufficiently. To indicate the value for PKPD modelling we have included more references and the following text in the discussion: “In addition, the killing rate as determined in in vitro rate-of-kill assays is a frequently-used parameter in dynamic PKPD models that aim to predict in vivo and clinical outcomes for infectious diseases such as malaria [25], TB [8] and other bacterial diseases [26-28].”

We have also included a sentence clarifying how rate-of-kill data could be useful for profiling combination treatment, but we believe it is outside the scope of this work to generate combination rate-of-kill data, we solely propose that this type of data has multiple uses.

Added sentence regarding combinations: “When assessing combination treatment, it may be valuable to determine the rate-of-kill of the combination as well as individual partner drugs, as some combinations may achieve a faster rate-of-kill than any of the partners individually (e.g. [29]). Such combinations would offer the potential of shorter treatment regimens.”

Specific comments:

-	A section on image analysis algorithm needs more clarifications.

o	Is image pre-processing performed?

We have amended to text to indicate that no pre-processing was performed (raw images were used).

- How was demarcation of host cell cytoplasm done?

We have expanded the image analysis section to provide more detail.

- Was there any size and/or intensity threshold used for identifying true parasites versus background (and/or host cells).

Yes, we have included the filters applied in the methods section.

- Does the algorithm only measure intracellular amastigote or trypomastigote as well?

The algorithm only detects parasites in the cytoplasm, this is clarified in the methods section.

- How were percentage of infected cells calculated?

This was calculated by quantifying the number of infected host cells (using “Select Population” building block that identifies host cells with one or more intracellular parasites) and expressing this as a percentage of total H9C2 cells in the image. The methods and results sections has been amended to clarify this.

- Approximately, what percentage of a 384 well plate was imaged for every time point?.

For each timepoint we acquired one image per well (850 x 630 µm, 535,500 µm^2^), and six wells per compound at each concentration. So, for an 8-point dose response curve, 48 wells were imaged, with a total area of ~26E6 µm^2^ (0.26 cm^2^). Total growth area of a 384 well plate is ~21.5 cm^2^, so in this example approximately 1.2% of the total growth area is imaged at every timepoint. The number of wells imaged per experiment varied depending on number compounds and concentrations tested. We have included the area covered per image in the methods section.

- What was the host cell monolayer confluence, were there specific sections of wells imaged, like the center of the well / edge?

The host cell monolayer was near confluency and the centre of the well was imaged. The methods section has been amended to clarify this.

Reviewer #2: yes to all

--------------------

<b>Results</b></br></br>

-Does the analysis presented match the analysis plan?</br> -Are the results clearly and completely presented?</br> -Are the figures (Tables, Images) of sufficient quality for clarity?</br></br>

Reviewer #1: General comments

-	Single concentration of compound was used to generate the time-course data. The concentrations used were arbitrary. Authors may have to explain the reasoning behind the concentration selected for rate of kill analysis.

As mentioned above we have now included data at multiple concentrations for each series.

- The EC50 data for this specific parasite in this cell line is needed. EC50 would be great starting point to compare compounds across multiple series.

We have included potencies determined using our dose-response RoK data for benznidazole, nifurtimox and fexinidazole sulfone. In terms of comparing between compounds we are representing the fastest RoK observed from multiple concentrations tested (at which no host cell toxicity was seen). This is now clarified in the text.

-	One possibility is to generate EC50 or EC90 for all the compounds using 10 point dose response. Use atleast 3 concentration 3X EC50, 10X EC50 and 25X EC50 for rate of kill for all compounds. This might help in interpreting data and also comparison across compounds become more meaningful.

We have now included plots for at least four concentrations for each compound series. For the NCEs (Figure 3), the chosen concentrations were guided by EC_50_s generated in our routine assays. The lowest concentration tested in RoK was always above the EC_50_ from the routine assay. We have expanded the text to make this clear.

-	It would be critical to generate rate of kill using varying concentration of compound. This would give more confidence, if the compounds show time or concentration dependent kill.

As explained above we have now included data for multiple concentrations.

-	Authors describe that the rate of kill experiments generated would help in PKPD analysis and Combination. But the data generated falls short of providing guidance for PKPD and combination, as the data generated is single point.

Please see comments above regarding multiple concentrations, PKPD modelling and combination approaches. The aim of this manuscript is to disclose a new assay to determine rate-of-kill profiles, and as such it makes sense for us to list potential applications of this type of data rather than providing data for each potential application. We believe that by expanding the start of the discussion we provide sufficient additional information regarding the value of this type of data for multiple purposes.

Specific comments:

- kindly clarify the timelines mentioned, for example, in lines 175 and 179, the authors mention at least four days and day 3 to 4 respectively, which I assume is the time post seeding in 384 well plates (from time 0 in Figure 1A) and not from time of infection. (similar to line 186 which clarifies this timeline clearly).

We have clarified this in the text.

-	Line 182: explain briefly, how the level of infection, i.e., cells infected calculated?

We have clarified this in the methods and the results section.

-	Lines 206-207: authors mention “we averaged data from six replicate wells for each condition to obtain data for a minimum of 2,000 host cells”. Does this mean each well with at least 2,000 host cells was considered a replicate with 6 such wells / replicates analyzed, or minimum 2000 cells were considered after combining 6 wells?

The combination of 6 wells results in at least 2,000 H9C2 cells being imaged, we have clarified this in the text.

-	Lines 209-210: How were the saturating concentrations for each compound determined? Is his based on a standard dose response assay – is it a multiple of EC50 or EC90. This is critical since all compounds have been tested only at a single concentration.

We have removed the saturating concentration statement and instead now show RoK curves for multiple concentrations.

-	For Figure 3. For new chemical matters it would be nice to show the amastigote over time graph (similar to Fig 2 B).

This has now been included (Figure 3B).

-	Fig 3: ES08 and MetRS have the fastest and second fastest rate of kill but still slower than benznidazole and nifurtimox (as seen in Fig.2). Authors can comment on how to use the data from this assay for the NCE series prioritization?

As commented above we have included an additional sentence on how and why rate-of-kill information can be included in compound triaging.

-	Fig. 3. All cpds were tested at 16.7 uM. It will be good to know the relative concentration to their respective EC50. This would help in understanding the Rate of kill pattern relative to the EC50.

As explained above the concentrations shown were selected from RoK profiles at at least 4 concentrations, and the concentration at which the fastest RoK was observed is shown. Combined, this data allows comparison of the profiles between series.

-	Lines 220-223 (and Fig. S3 and Movie S2): The parasites numbers are cleared to the limit of detection in nifurtimox treatment as show in Fig 2 in the current assay format. A similar Rate of Kill movie for Posaconazole and Benznidazole would be highly beneficial. It will be interesting to see, if Posaconazole arrests replication (static) or even fails to block replication visually.

We have added movies for benznidazole and posaconazole. Posaconazole is able to reduce parasite levels albeit slower than benznidazole and nifurtimox and after allowing an initial phase of parasite growth.

Reviewer #2: yes to all (except missing caption Table S2; see below)

--------------------

<b>Conclusions</b></br></br>

-Are the conclusions supported by the data presented?</br> -Are the limitations of analysis clearly described?</br> -Do the authors discuss how these data can be helpful to advance our understanding of the topic under study?</br> -Is public health relevance addressed?</br></br>

Reviewer #1: General comments:

Authors have described the live monitoring assay, which specifically address the drawbacks of currently available RoK experiments such as low time resolution and track same cell population over time. But the usefulness of RoK using single concentration of compounds is insufficient to contribute to compound prioritization, comparison across different series of cpds and also within the same series. The behavior of kill pattern might change depending on the concentration of individual compound. The interpretation of same series having same pattern might be too strong for the data presented.

Specific comments

-	Lines 294 to 301: Oxaboroles have shown be fast acting against T. cruzi by Moraes et. al. (as discussed by authors) and other trypanosomes. Moraes et al publication used multiple concentrations and showed fast kill. Since the current study is only using single concentration , comparison is challenging and may be less meaningful.

We have now included data for 4 concentrations of the oxaborole compound (SCYX-6759 / AN4169). While Moraes et al see a maximum killing rate at low micromolar concentrations (~2.5µM), we see much slower killing at higher concentration (5.5 and 16.6µM), indicating that there is a substantial difference. It is possible that at higher concentrations we would see faster killing.

-	Lines 403 to 406 - Fig. S1: also include information on percentage of host cells and parasites expressing the fluorescent tags.

We have expanded Figure S1 to include % of E2-Crimson positive epimastigotes and trypomastigotes.

-	A live imaging assay was recently published as briefly mentioned by the authors as well (Fesser AF et al. PLoS Negl Trop Dis 14(7): e0008487). The authors assay is significantly improved compared to the previous published data, however, the authors do not discuss their data in comparison this recently published live imaging assay.

Fesser et al. only report data for benznidazole and posaconazole, and while no kill rates are generated their data appears comparable for these compounds, as mentioned in the discussion (lines 308 – 309 in tracked changes document). We also outline the key advantages of our assay over other assays, including the assay from Fesser et al on lines 301 – 305. We have expanded this last section to clarify the differences more explicitly.

-	One of the conclusion from the assay is that the compounds from the same series behave similarly in the live imaging assay. Although it is looking similar for NCEs, it may be advisible to test 1-2 more azoles and check, if they behave similar to Posaconazole. (as tested by authors in their previous rate-of-kill assay in MacLean LM et al. PLoS Negl Trop Dis 10(4): e0004584).

We have included multiple compounds from multiple series to support the point that compounds from the same series/mode of action have similar RoK profiles, so we feel it is not essential to generate this additional data to support our conclusion.

Reviewer #2: yes to all

--------------------

<b>Editorial and Data Presentation Modifications?</b></br><br/> Use this section for editorial suggestions as well as relatively minor modifications of existing data that would enhance clarity. If the only modifications needed are minor and/or editorial, you may wish to recommend “Minor Revision” or “Accept”.

Reviewer #1: None

Reviewer #2: 1. Data is the plural of datum. Therefore "allow" (Line 26), "these" (Line 187), "show" (Line 210), "indicate" (line 307).

We have corrected these.

2. Table S1. The caption is missing. Please clarify in the caption whether "Slope" refers to the maximal slope and how the "Lag phase" was calculated.

Figure S1 caption is now included. Method to determine slope and lag phase are described in methods.

--------------------

<b>Summary and General Comments</b></br></br> Use this section to provide overall comments, discuss strengths/weaknesses of the study, novelty, significance, general execution and scholarship. You may also include additional comments for the author, including concerns about dual publication, research ethics, or publication ethics. If requesting major revision, please articulate the new experiments that are needed.

Reviewer #1: The authors have developed a live imaging assay to determine the rate of kill of compounds against the Chagas T. cruzi intracellular parasite stage. For the assay, fluorescent parasites and host cell numbers were tracked and quantified over time with a predominant focus on parasites as the host cells do not replicate in the measured time. Using the assay, clinical compounds were profiled at a single concentration along with a few more previous published new chemical entities for comparison. The assay is robust, reproducible with valuable data on rate of kill of compounds.

But the interpretations are made based on limited single concentration of compound testing could be over-interpreting the data obtained.

Reviewer #2: Svensen et al. describe an elegant new in vitro assay for monitoring the action of test compounds against intracellular T. cruzi in real time and for quantifying the rate-of-kill. There are several improvements as compared to previously published assays (expression of nucleus-restricted GFP in the host cells, reduced level of background fluorescence). The method and the results are described very clearly, and thus this manuscript represents a substantial advance in the field of drug discovery for Chagas disease.

--------------------

PLOS authors have the option to publish the peer review history of their article (<a href="<https://journals.plos.org/plosntds/s/editorial-and-peer-review-process#loc-peer-review-history>" target="_blank">what does this mean?</a>). If published, this will include your full peer review and any attached files.<br><br>

If you choose “no”, your identity will remain anonymous but your review may still be made public.<br><br>

<b>Do you want your identity to be public for this peer review?</b> For information about this choice, including consent withdrawal, please see our <a href="<https://www.plos.org/privacy-policy>" target="_blank">Privacy Policy</a>.

Reviewer #1: No

Reviewer #2: No
